# Ischaemic stroke and intracerebral haemorrhage in the post-acute phase after myocardial infarction: a nationwide cohort study in Sweden

**DOI:** 10.1093/esj/aakag138

**Published:** 2026-11-12

**Authors:** Putu Ayuwidia Ekaputri, Adam Viktorisson, Petra Redfors, Margrét Leósdóttir, Katharina S Sunnerhagen

**Affiliations:** Department of Clinical Neuroscience, Institute of Neuroscience and Physiology, Sahlgrenska Academy, University of Gothenburg, Gothenburg, Sweden; Department of Rehabilitation Medicine, Sahlgrenska University Hospital, Gothenburg, Sweden; Department of Clinical Neuroscience, Institute of Neuroscience and Physiology, Sahlgrenska Academy, University of Gothenburg, Gothenburg, Sweden; Department of Rehabilitation Medicine, Sahlgrenska University Hospital, Gothenburg, Sweden; Department of Clinical Neuroscience, Institute of Neuroscience and Physiology, Sahlgrenska Academy, University of Gothenburg, Gothenburg, Sweden; Department of Neurology, Sahlgrenska University Hospital, Gothenburg, Sweden; Department of Clinical Sciences Malmö, Lund University, Malmö Sweden; Department of Cardiology, Skåne University Hospital, Malmö, Sweden; Department of Clinical Neuroscience, Institute of Neuroscience and Physiology, Sahlgrenska Academy, University of Gothenburg, Gothenburg, Sweden; Department of Rehabilitation Medicine, Sahlgrenska University Hospital, Gothenburg, Sweden

**Keywords:** intracerebral haemorrhage, ischaemic stroke, myocardial infarction follow-up, myocardial infarction survivor, stroke incidence, SWEDEHEART

## Abstract

**Introduction:**

Patients who survive myocardial infarction (MI) remain at risk of stroke in the post-acute MI-phase, after discharge. We investigated the incidence of ischaemic stroke and intracerebral haemorrhage in MI survivors attending early follow-up.

**Patients and methods:**

We included MI survivors from the SWEDEHEART registry linked to national health registers, who attended a cardiac rehabilitation visit 6–10 weeks post-MI between 2005 and 2020 and followed them until 31 December 2021. Fine–Grey competing-risk regression was used to assess associations between clinical factors and stroke outcomes.

**Results:**

Among 85,360 MI survivors, 4275 patients experienced ischaemic stroke or intracerebral haemorrhage during follow-up. The incidence of ischaemic stroke was highest during the first year after MI (~1.1%) and declined thereafter, while intracerebral haemorrhage remained rare throughout follow-up. Reduced left ventricular ejection fraction (LVEF) was independently associated with higher ischaemic stroke risk. Compared with normal LVEF, patients with moderately reduced LVEF (30%–39%) had an sHR of 1.47 (95% CI, 1.14–1.90) at 1 year and 1.47 (95% CI, 1.25–1.74) at 5 years, while those with severely reduced LVEF (<30%) had an sHR of 1.62 (95% CI, 1.11–2.35) at 1 year and 1.32 (95% CI, 1.02–1.71) at 5 years. Atrial fibrillation (AF) was strongly and independently associated with ischaemic stroke risk (sHR 2.21; 95% CI, 1.70–2.88 at 1 year; sHR 1.54; 95% CI, 1.28–1.84 at 5 years). While oral anticoagulation alone was not associated with reduced stroke risk, it was associated with significantly lower ischaemic stroke risk among patients with AF (AF × oral anticoagulant interaction sHR 0.45; 95% CI, 0.27–0.75 at 1 year).

**Conclusion:**

Stroke risk after MI is highest during the first year and is strongly influenced by AF presence and reduced LVEF. These findings highlight the importance of early risk stratification and targeted prevention strategies after MI.

**Graphical abstract:**
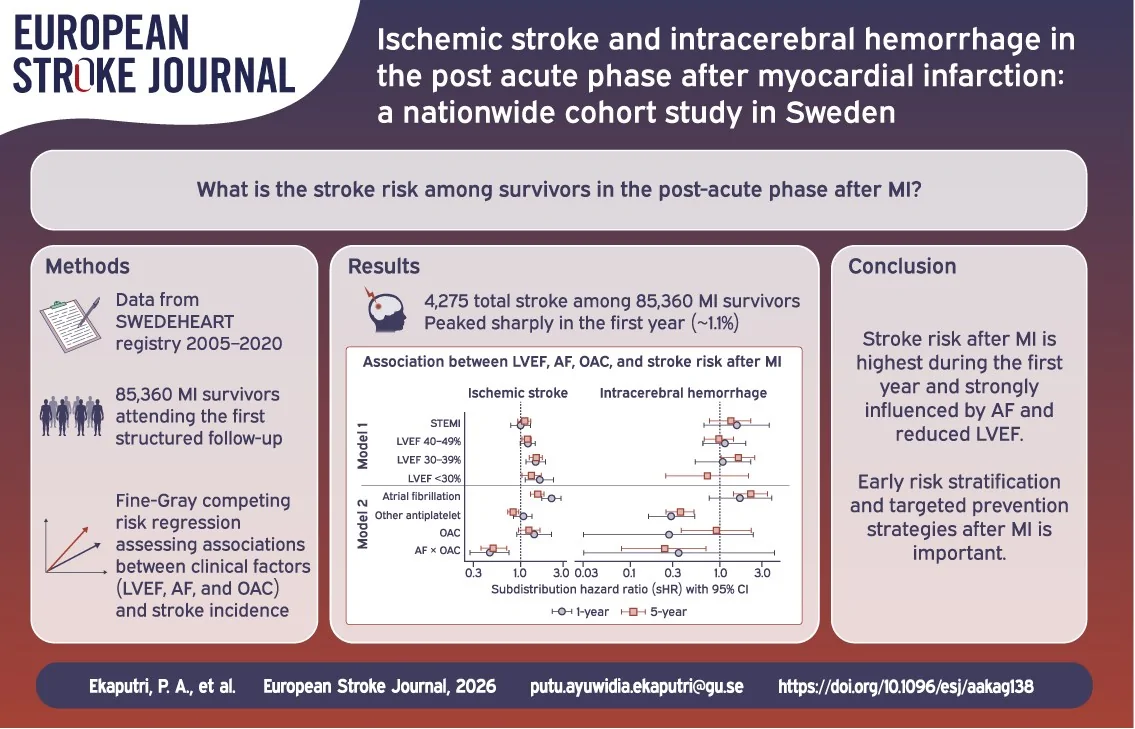

## Introduction

Stroke is a serious and potentially disabling complication of cardiovascular disease. Patients with acute myocardial infarction (MI) are at increased risk of suffering a stroke, with 1-year incidence for ischaemic stroke estimated between 2% and 4% in registry-based cohorts.^1–3^ Prior studies have identified key baseline risk factors such as age, atrial fibrillation (AF), diabetes and heart failure, and have shown a decline in stroke incidence over recent decades because of improvements in reperfusion therapy, medical treatment and secondary prevention after MI.^1^^,^^2^^,^^4^^,^^5^

Most existing studies have focused on the acute hospitalisation period or the first 30 days after MI, when stroke risk is highest.^2^^,^^3^ During this early period, acute thrombotic and inflammatory processes, together with LV dysfunction and blood stasis, may contribute to stroke risk.^6^^,^^7^ However, more recent evidence shows that stroke risk remains elevated beyond 30 days and up to 12 weeks after MI.^8^ However, these studies have generally followed patients from the time of MI, making it difficult to distinguish risk during the acute period from risk among patients who survive and reach the post-acute outpatient phase. In this setting, ongoing cardiac dysfunction and thromboembolic risk may remain relevant to subsequent stroke. Reduced left ventricular ejection fraction (LVEF) may contribute to thromboembolic risk through left ventricular (LV) dysfunction, blood stasis and LV thrombus formation.^6^^,^^9^ Conversely, antithrombotic treatment may modify this risk; oral anticoagulant (OAC) is established for stroke prevention in patients with AF and is used in selected patients after MI,^10^ including those with LV thrombus,^9^ but its association with subsequent stroke risk in patients who have reached the post-acute phase after MI is less well characterised.

To improve the understanding of stroke risk in the post-acute MI phase, where secondary prevention strategies may be most effectively targeted, we conducted a study using a national cohort of patients with MI who attended a structured outpatient follow-up visit 6–10 weeks after the acute event. We aimed to estimate stroke incidence in patients who attended follow-up accounting for death and competing stroke subtypes as competing events. Furthermore, we aimed to investigate the association between LVEF and OAC use with stroke risk within the patients who attend the post-MI follow-up.

## Patients and methods

### Study design and data sources

This nationwide cohort study used data from the Swedish Web-system for Enhancement and Development of Evidence-based Care in Heart Disease Evaluated According to Recommended Therapies (SWEDEHEART) registry (https://www.ucr.uu.se/en/). Myocardial infarction cases were identified from the cardiac rehabilitation (CR) component of the SWEDEHEART registry (SWEDEHEART-CR). The registry represents 75%–80% of all patients under 80 years of age who are discharged after MI and subsequently attend CR during the first post-MI year,^11^ with the first follow-up happened around 6–10 weeks after hospital discharge. This visit served as the study baseline. SWEDEHEART-CR only captures patients who return for follow-up; therefore, the cohort inherently reflects individuals who have survived the acute phase of MI and entered the post-acute management phase.

Baseline characteristics of the subjects were obtained from both the acute hospitalisation and the follow-up visit. Acute-phase characteristic included type of MI (ST elevation MI/non-ST elevation MI) and LVEF, categorised according to SWEDEHEART as: normal (≥50%), slightly reduced (40%–49%), moderately reduced (30%–39%) and severely reduced (<30%). Reperfusion (percutaneous coronary intervention or coronary artery bypass grafting) or thrombolytic therapy during the acute phase was also included.

Comorbidities were identified using the National Patient Register (NPR), which includes all inpatient and specialist outpatient diagnoses in Sweden. Diagnoses recorded before the follow-up date (with a + 7-day margin) were included. Mortality rates were obtained from the Swedish Cause of Death Register. All datasets were linked and pseudonymised by the Swedish National Board of Health and Welfare.

Follow-up medication use included OACs, acetylsalicylic acid (ASA), angiotensin-converting enzyme (ACE) inhibitors/angiotensin receptor blockers (ARBs), beta-blockers, statins, calcium antagonists, diuretics, insulin, digitalis and nitrates. Comorbidities were identified from NPR diagnoses recorded prior to follow-up (+7 days) and were defined using ICD-10 codes, as detailed in Table S1, including AF, diabetes, hypertension, hyperlipidaemia, alcohol overconsumption, overweight, arteries disease, heart failure and renal dysfunction.

#### Assessment of anticoagulant use

Oral anticoagulant use after MI was assessed using data from SWEDEHEART-CR and the Swedish Prescribed Drug Register. Data on patients prescribed OAC by the treating cardiologists at the 6- to 10-week follow-up visit were obtained from SWEDEHEART-CR, and dispensation within ± 30 days (“0 months”) was verified using ATC codes for vitamin K antagonists (B01AA), direct thrombin inhibitors (B01AE) and factor Xa inhibitors (B01AF). Oral anticoagulant persistence was evaluated at 3 months, 6 months, 1 year and 5 years. Comparisons were made by AF status and LVEF groups to assess differences in uptake and long-term adherence.

### Study population

We included patients with MI treated between 1 January 2005 and 31 December 2020, who attended the 6- to 10-week follow-up visit. The visit occurred at a median of 59 days (range 19–100 days) after the index MI event. We excluded patients who were under 18 years of age at the time of MI, attended follow-up later than 100 days post-discharge or had a prior stroke before follow-up.

### Outcomes

The primary outcomes were ischaemic stroke and intracerebral haemorrhage, identified using ICD-10 codes (Table S1) reported to the National Patient Register or registered as cause of death in the Swedish Cause of Death Register. Only patients whose first recorded stroke in the available registry data occurred after the index MI follow-up date were included as stroke cases. Patients were followed until stroke, death or 31 December 2021. Each stroke subtype was analysed separately, treating the other subtype and death as competing events.

### Statistical analysis

#### Descriptive analysis

Baseline characteristics were summarised separately for patients who developed stroke during follow-up and for those who did not. Continuous variables were presented as means with SD, and categorical variables were presented as counts and percentages. Missing values were reported explicitly for each variable. Between-group differences were tested using chi-square tests for categorical variables and *t*-tests for continuous variables. A *P*-value < .05 was considered statistically significant.

#### Stroke incidence

Annual stroke incidence rates were calculated as the number of events per 100 MI patients at risk for each follow-up year after the index visit. Incidence rates were estimated separately for total stroke and for ischaemic and haemorrhagic subtypes. Rates were derived using the number of new stroke events within each year divided by the number of patients still at risk at the beginning of that year, with deaths and prior strokes excluded from the denominator. Stroke incidence was expressed as events per 100 patients per year, and trends were plotted across the 15-year follow-up period. Kaplan–Meier survival curves were generated for exploratory visualisation, treating death as censoring. Curves were stratified by age, sex, LVEF, AF and OAC use, and plotted separately for ischaemic and intracerebral haemorrhage.

#### Competing risk analysis

We used Fine–Grey subdistribution hazard models to estimate cause-specific risk accounting for competing risk. This analysis directly models the subdistribution hazard, linking covariates to the cumulative incidence function by retaining individuals who experience competing events in the risk set with appropriate weighting. The cumulative incidence function provides estimates of absolute risk at prespecified time points in the presence of competing events. Ischaemic stroke and intracerebral haemorrhages were analysed separately, treating the alternate stroke subtype and death as competing risks. Follow-up was censored at 1 year and 5 years.

We specified 2 models:

1. Model 1 investigates the relationship between acute-phase LVEF and subsequent stroke risk, adjusting for relevant confounders. LVEF was assessed during the index MI hospitalisation and was included to investigate whether cardiac function during the acute phase was associated with subsequent stroke risk among MI survivors. Associations were adjusted for age, sex, infarct type, reperfusion therapy and comorbidities (AF, diabetes, hypertension, dyslipidaemia, alcohol overconsumption, overweight, peripheral artery disease, heart failure and renal dysfunction).
2. Model 2 investigates the relation between AF, OAC and other antiplatelets (ASA and clopidogrel), adjusting for relevant confounders. Associations were adjusted for age, sex, infarct type, reperfusion therapy, other comorbidities (diabetes, hypertension, dyslipidaemia, alcohol overconsumption, overweight, arteries disease, heart failure and renal dysfunction) and other medications recorded at the 6- to 10-week follow-up visit after MI (other antiplatelets, ASA, ACE inhibitors (ACEi)/ARBs, beta-blockers, statins, calcium antagonists, diuretics, insulin, digitalis, nitrates). The following interaction terms were also included in the model: AF × OAC, heart failure × beta-blocker use and hypertension × ACEi/ARB use.

Age was modelled per 10-year increase. Results are reported as adjusted subdistribution hazard ratios (sHRs) with 95% CIs. Missing data were handled using complete-case analysis. Individuals with missing values for any covariate included in the Fine–Grey models were excluded from the corresponding analysis, and no imputation was performed. Thus, each model was fitted using individuals with complete data for all variables included in that model. Of the total cohort of 85,360 patients, 66,057 (77.4%) had complete data for all covariates included in Model 1 and 63,261 (74.1%) had complete data for all covariates included in Model 2. These analytic sample sizes were the same for the 1- and 5-year analyses and for ischaemic and haemorrhagic stroke outcomes. For stratified Kaplan–Meier analyses, patients with missing data for the respective stratification variable were excluded. Therefore, the number of patients included in these analyses may differ from that in the multivariable Fine–Grey models. All analyses were performed using RStudio (Version 2025.05.1 + 513) with the *cmprsk*, *riskRegression* and survival packages.

## Results

Between 1 January 2005 and 31 December 2020, a total of 94,620 patients with a history of MI attended follow-up visit recorded in the SWEDEHEART-CR registry. Among these, 85,411 patients attended the 6- to 10-week follow-up visit within 100 days after the MI admission date. After excluding patients under 18 years of age, the study population consisted of 85,360 adult patients. During subsequent study follow-up until 31 December 2021, 4275 patients experienced a stroke, of whom 3760 (88.0%) had an ischaemic stroke and 515 (12.0%) had an intracerebral haemorrhage.

Patients with MI who developed stroke were older (mean 66.8 years, SD 7.5 vs 63.9 years, SD 9.0) and included a slightly higher proportion of women (26.4% vs 25.6%). They also exhibited poorer LV function and were less frequently treated with reperfusion therapy at the index MI (Table 1). Cardiovascular risk factors and comorbidities were also more prevalent in this group, particularly diabetes, AF, hypertension, peripheral artery disease, heart failure and renal dysfunction. Patients with MI who later developed stroke had higher use of OAC, other antiplatelet agents, beta-blockers, calcium antagonists, insulin, digitalis, diuretics and nitrates at baseline, while statins and ASA use was slightly less frequent compared to patients who did not develop stroke.

**Table 1:** Patient characteristics at the 6- to 10-week follow-up visit after myocardial infarction, stratified by stroke events.

Variable	Stroke (*n* = 4,275)			No stroke (*n* = 81,085)			*P*-value
	*n*	%	Missing, *n*	*n*	%	Missing, *n*	
***Demographics***							
**Age, mean (SD), years**	66.8 (±7.5)		—	63.9 (±9.0)		—	<.001
**Male sex**	3,148	73.6	—	60,313	74.4	—	.001
***Index MI characteristics***							
**STEMI**	1,119	36.2	1,188	28,451	39.9	9,882	.001
**Left ventricular function**			702			9,963	<.001
**Normal (≥50%)**	2,089	58.5		47,193	66.4		
**Slightly reduced (40%–49%)**	799	22.4		14,919	21.0		
**Moderately reduced (30%–39%)**	527	14.7		6,987	9.8		
**Severely reduced (<30%)**	158	4.4		2,023	2.8		
**Reperfusion therapy**			0			8	<.001
**Thrombolysis**	108	2.5		1,700	2.1		
**Primary PCI**	1,374	32.1		29,603	36.5		
**Acute CABG**	11	0.3		80	0.1		
***Lifestyle***							
**Smoking status**			39			559	<.001
**Never smoker**	1,314	31.0		27,790	34.5		
**Former smoker (>1 month)**	2,351	55.5		44,504	55.3		
**Current smoker**	571	13.5		8,232	10.2		
***Discharge medications***							
**ACE inhibitor or ARB**	3,518	82.8	25	66,112	81.9	368	.156
**OAC**			31			494	<.001
**Warfarin**	355	8.4		3,867	4.8		
**Ximelagatran**	—	—		2	0.0		
**Dabigatran**	18	0.4		320	0.4		
**Rivaroxaban**	24	0.6		602	0.7		
**Apixaban**	61	1.4		1,977	2.5		
**Edoxaban**	2	0.0		34	0.0		
**Aspirin (ASA)**	3,920	92.3	28	75,476	93.6	406	.002
**Other antiplatelet agent**			26			389	<.001
**Clopidogrel**	2,462	57.6		30,532	37.6		
**Ticagrelor**	950	22.2		40,175	49.6		
**Prasugrel**	59	1.4		1,169	1.4		
**Ticlopidine**	23	0.5		143	0.2		
**Beta-blocker**	3,824	90.0	28	70,395	87.2	379	<.001
**Calcium antagonist**	917	21.6	29	12,694	15.7	439	<.001
**Insulin**	613	14.5	33	6,621	8.2	439	<.001
**Digitalis**	106	2.5	31	720	0.9	436	<.001
**Diuretic**	1,109	26.1	31	12,701	15.7	436	<.001
**Statin**	3,968	93.4	27	77,324	95.8	409	<.001
**Nitrates**	461	10.9	35	5,203	6.5	546	<.001
***Comorbidities***							
**Diabetes mellitus**	1,320	30.9	0	16,740	20.6	0	<.001
**Atrial fibrillation**	686	16.0	0	8,083	10.0	0	<.001
**Hypertension**	2,782	65.1	0	44,953	55.4	0	<.001
**Hyperlipidaemia**	1,644	38.5	0	28,343	35.0	0	<.001
**Alcohol overconsumption**	499	11.7	0	9,828	12.1	0	.400
**Overweight**	227	5.3	0	4,493	5.5	0	.537
**Arteries disease**	448	10.5	0	4,666	5.8	0	<.001
**Heart failure**	852	19.9	0	11,631	14.3	0	<.001
**Renal dysfunction**	309	7.2	0	4,145	5.1	0	<.001

Abbreviations: ACE = angiotensin-converting enzyme; ARB = angiotensin receptor blocker; ASA = acetylsalicylic acid; CABG = coronary artery bypass grafting; MI = myocardial infarction; NSTEMI = non–ST-elevation myocardial infarction; OAC = oral anticoagulant; PCI = percutaneous coronary intervention; STEMI = ST-elevation myocardial infarction.

Data are presented as mean (SD) for continuous variables and *n* (%) for categorical variables. Missing values are shown at the variable level only.

### Stroke incidence after myocardial infarction

When presented per 100 MI patients (Figure 1), ischaemic stroke incidence peaked sharply in the first year (~1.1%), then steadily declined over time, while intracerebral haemorrhage incidence remained consistently low throughout follow-up (~0.05%–0.13% per year).

**Figure 1:**
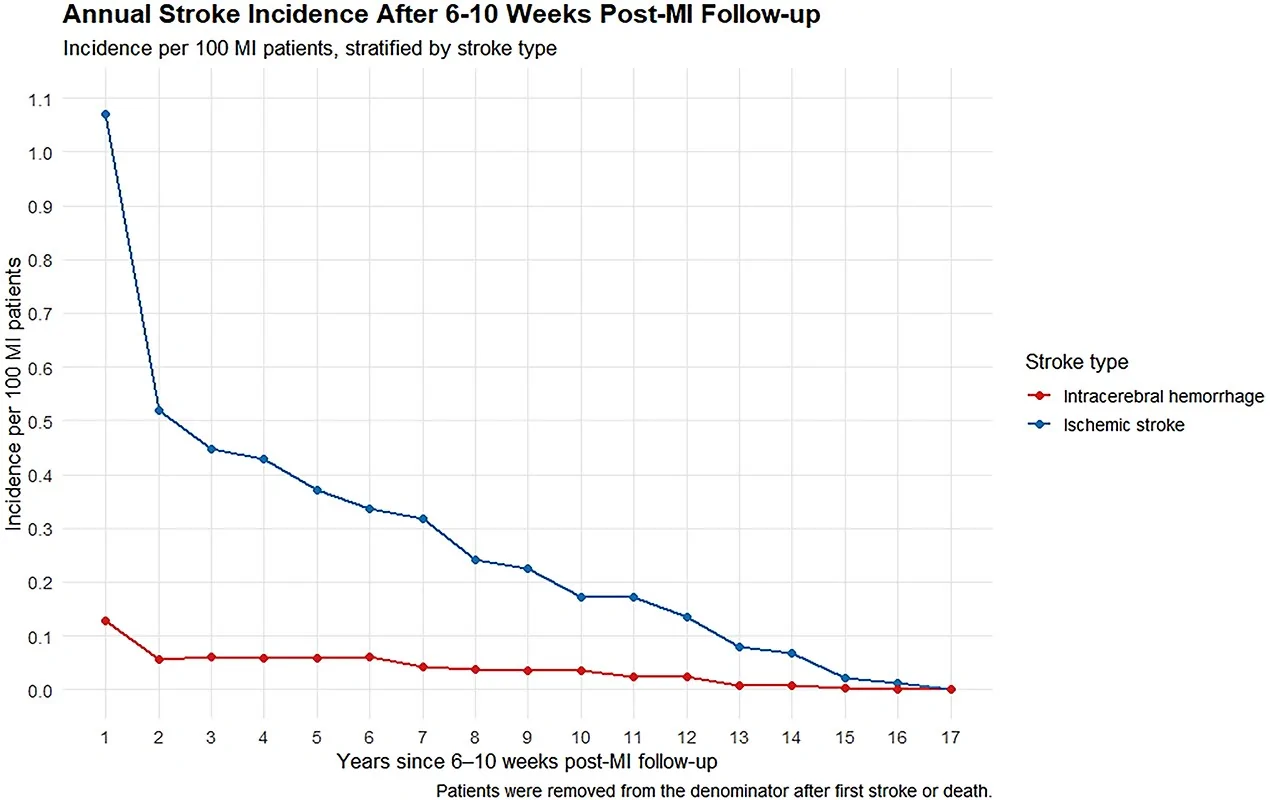
Annual stroke incidence per 100 patients following myocardial infarction, stratified by stroke type. Rates shown separately for ischaemic and intracerebral haemorrhage, by years since the 6- to 10-week post-MI follow-up visit. Abbreviation: MI = myocardial infarction.

The crude cumulative incidence of haemorrhagic and ischaemic stroke is presented in Figure S1 and S2. The cumulative incidence of ischaemic stroke increased steadily over time, approaching more than 5% at 5-year follow-up in patients older than 65 years, with AF history, and those with severely reduced LVEF (<30%). Men and women showed similar cumulative incidence (*P* = .20). Patients with AF after MI had a markedly higher cumulative incidence compared with those without AF (*P* < .0001), and a clear increasing incidence was observed as LVEF decreased (*P* < .0001) and as age increased (*P* < .0001). Patients using OAC also had higher cumulative incidence of ischaemic stroke (*P* < .0001).

The cumulative incidence of intracerebral haemorrhage was low throughout follow-up, remaining below 2% even at long-term follow-up. For intracerebral haemorrhage, there were no significant differences between men and women (*P* = .74), or LVEF groups (*P* = .33). In contrast, patients with AF after MI (*P* < .0001), and patients older than 65 years (*P* < .0001) had higher incidence of intracerebral haemorrhage. Furthermore, OAC users also showed a higher incidence of intracerebral haemorrhage compared with non-users (*P* = .0033).

### LVEF and stroke risk

Compared with patients with normal LVEF (≥50%), those with moderately reduced LVEF (30%–39%) had an adjusted subdistribution hazard ratio (sHR) of 1.47 (95% CI, 1.14–1.90; *P* = .003) at 1 year and 1.47 (95% CI, 1.25–1.74; *P* < .001) at 5 years for ischaemic stroke (Table 2, Model 1). Patients with severely reduced LVEF (<30%) had the highest risk, with an sHR of 1.62 (95% CI, 1.11–2.35; *P* = .012) at 1 year and 1.32 (95% CI, 1.02–1.71; *P* = .035) at 5 years. For intracerebral haemorrhage, only moderately reduced LVEF (30%–39%) was associated with increased risk at 5 years (sHR 1.60; 95% CI, 1.03–2.49, *P* = .037), with no significant associations at 1 year (Table 2, Model 1).

**Table 2:** Competing risk analysis of association between left ventricular ejection fraction and stroke risk after myocardial infarction.

Model 1	1 year				5 years			
	Ischaemic stroke		Intracerebral haemorrhage		Ischaemic stroke		Intracerebral haemorrhage	
	**sHR (95% CI)**	***P*-value**	**sHR (95% CI)**	***P*-value**	**sHR (95% CI)**	***P*-value**	**sHR (95% CI)**	***P*-value**
**Infarct type (STEMI)**	1.00 (0.77–1.31)	.970	1.53 (0.66–3.58)	.320	1.10 (0.93–1.29)	.280	1.31 (0.77–.24)	.320
**LVEF: Slightly reduced (40%–49%)**	1.21 (0.99–1.48)	.060	1.12 (0.64–1.97)	.700	1.17 (1.03–1.32)	.018	0.98 (0.67–1.43)	.910
**LVEF: Moderately reduced (30%–39%)**	1.47 (1.14–1.90)	.003	1.09 (0.53–2.23)	.820	1.47 (1.25–1.74)	<.001	1.60 (1.03–2.49)	.037
**LVEF: Severely reduced (<30%)**	1.62 (1.11–2.35)	.012	N/A*	N/A*	1.32 (1.02–1.71)	.035	0.73 (0.25–2.11)	.560

Abbreviations: LVEF = left ventricular ejection fraction; MI = myocardial infarction; sHR = subdistribution hazard ratios; STEMI = ST-elevation myocardial infarction.

The analytic sample for Model 1 comprised 66,057 patients with complete data for all included covariates (77.4% of the total cohort of 85,360). Adjusted sHRs and 95% CIs for ischaemic stroke and intracerebral haemorrhage derived from Fine–Grey competing risk models, accounting for death as a competing event. Estimates are shown for 1-year and 5-year follow-up. Model 1 was adjusted for age, sex, reperfusion therapy and comorbidities (atrial fibrillation, diabetes, hypertension, dyslipidaemia, alcohol overconsumption, overweight, arterial disease, heart failure and renal dysfunction). The association with infarct type (STEMI vs non-STEMI) is additionally reported. *No subjects with severely reduced LVEF experienced an intracerebral haemorrhage within 1 year after the first MI follow-up.

### Atrial fibrillation, OAC and stroke risk

Atrial fibrillation was associated with a higher risk of ischaemic stroke, with an sHR of 2.21 (95% CI, 1.70–2.88; *P* < .001) at 1 year and 1.54 (95% CI, 1.28–1.84; *P* < .001) at 5 years (Table 3, Model 2). Oral anticoagulant use alone was not significantly associated with ischaemic stroke (sHR 1.41, 95% CI, 0.90–2.22; *P* = .130 at 1 year; 1.25, 95% CI, 0.94–1.66; *P* = .120 at 5 years). However, the AF × OAC interaction indicated that the association between OAC use and ischaemic stroke differed by AF status. The relative association of OAC use with ischaemic stroke was lower among patients with AF than among those without AF (interaction sHR 0.45; 95% CI, 0.27–0.75; *P* = .002 at 1 year and sHR 0.50; 95% CI, 0.36–0.71; *P* < .001 at 5 years).

**Table 3:** Competing risk analysis of association between atrial fibrillation, antithrombotic therapy and stroke risk after myocardial infarction.

Model 2	1 year				5 years			
	Ischaemic stroke		Intracerebral haemorrhage		Ischaemic stroke		Intracerebral haemorrhage	
	**sHR (95% CI)**	***P*-value**	**sHR (95% CI)**	***P*-value**	**sHR (95% CI)**	***P*-value**	**sHR (95% CI)**	***P*-value**
**Atrial fibrillation**	2.21 (1.70–2.88)	<.001	1.70 (0.76–3.80)	.190	1.54 (1.28–1.84)	<.001	2.23 (1.44–3.46)	<.001
**Other antiplatelet (eg, clopidogrel)**	1.05 (0.82–1.35)	.700	0.29 (0.16–0.53)	<.001	0.83 (0.72–0.97)	.017	0.36 (0.25–0.52)	<.001
**OAC**	1.41 (0.90–2.22)	.130	0.27 (0.03–2.37)	.240	1.25 (0.94–1.66)	.120	0.91 (0.37–2.28)	.850
**AF × OAC**	0.45 (0.27–0.75)	.002	0.34 (0.03–4.11)	.390	0.50 (0.36–0.71)	<.001	0.24 (0.08–0.70)	.009

Abbreviations: ACE = angiotensin-converting enzyme; AF = atrial fibrillation; ARB = angiotensin receptor blocker; ASA = acetylsalicylic acid; MI = myocardial infarction; OAC = oral anticoagulant; sHR = subdistribution hazard ratios.

The analytic sample for Model 2 comprised 63,261 patients with complete data for all included covariates (74.1% of the total cohort of 85,360). Adjusted sHRs and 95% CIs for ischaemic stroke and intracerebral haemorrhage derived from Fine–Grey competing risk models, accounting for death as a competing event. Estimates are shown for 1-year and 5-year follow-up. Model 2 was adjusted for age, sex, infarct type, reperfusion therapy, other comorbidities (diabetes, hypertension, dyslipidaemia, alcohol overconsumption, overweight, arteries disease, heart failure and renal dysfunction) and other medications recorded at the first follow-up visit after MI (other antiplatelets, ASA, ACE inhibitors/ARBs, beta-blockers, statins, calcium antagonists, diuretics, insulin, digitalis, nitrates).

For intracerebral haemorrhage, AF was not significantly associated with stroke risk at 1 year (sHR 1.70; 95% CI, 0.76–3.80; *P* = .190) but was associated with a higher risk at 5 years (sHR 2.23; 95% CI, 1.44–3.46; *P* < .001). As for ischaemic stroke, OAC use alone was not significantly associated with intracerebral haemorrhage (sHR 0.27; 95% CI, 0.03–2.37 at 1 year; *P* = .240 and sHR 0.91; 95% CI, 0.37–2.28; *P* = .850 at 5 years). The AF × OAC interaction was associated with a lower risk of intracerebral haemorrhage with OAC use in patients with AF than in patients without AF at 5 years (sHR 0.24; 95% CI, 0.08–0.70; *P* = .009), but this was not statistically significant at 1 year (sHR 0.34; 95% CI, 0.03–4.11; *P* = .390). Antiplatelet therapy was associated with a lower risk of intracerebral haemorrhage at both 1 year (sHR 0.29; 95% CI, 0.16–0.53; *P* < .001) and 5 years (sHR 0.36; 95% CI, 0.25–0.52; *P* < .001) (Table 3, Model 2).

Oral anticoagulant was primarily prescribed to patients with AF, with 68.3% of anticoagulant users having AF compared to only 4.8% among non-users (Table S2). Oral anticoagulant users were generally older, with nearly 50% aged 71 or older, compared to 27.4% in the non-user group. The use of OAC also increased with higher CHA₂DS₂-VASc scores: 49.8% of users had a score ≥ 4 compared to 26.1% among non-users. Regarding cardiac function, moderate-to-severely reduced LVEF (≤39%) was more common among OAC users (27.0% vs 11.4%). Patterns of OAC use differed according to clinical indication and time since MI. Among patients with AF, two-thirds (67.8%) of those recorded as receiving OAC therapy at the 6- to 10-week follow-up also had a corresponding prescription dispensed in the national drug register, and 59.1% remained on treatment after 1 year (Table S3). In contrast, persistence was lower among patients without AF (35.5% at 1 year). A similar pattern was observed for patients with reduced LVEF, in whom 77.4% had collected OAC medication at baseline and 63.1% continued treatment at 1 year, compared with 52.9% among those with normal LVEF (≥50%).

## Discussion

This national cohort study evaluated the incidence and predictors of ischaemic stroke and intracerebral haemorrhage among 85,360 patients with MI who had survived the acute phase and attended a structured outpatient follow-up visit approximately 6–10 weeks after discharge. By focusing on this clinically stable post-MI population, our study provides insights into residual stroke risk beyond the immediate post-hospitalisation phase. During the first year of follow-up after MI, stroke incidence was approximately 1.2%, comprising about 1.1% ischaemic stroke and 0.13% intracerebral haemorrhage. Previous studies reported ischaemic stroke incidence of 2.1% at 30 days^3^ and 4.1% at 1 year^12^ after MI, with declining rates attributed to improvements in reperfusion therapy and secondary prevention. Although the incidence in our clinically stable population was lower, our findings indicate that clinically relevant stroke risk persist after stabilisation. Intracerebral haemorrhage remained uncommon, consistent with previous evidence of declining haemorrhagic stroke rates after MI.^13^

Oral anticoagulant is well-established after MI in patients with AF or LV thrombus.^9^^,^^14^ Even in the absence of AF, patients with reduced LVEF may remain at elevated risk of thromboembolic events,^15–18^ providing a rationale for earlier consideration of OAC based on pathophysiological considerations and observational evidence.^19^ However, randomised controlled trials and recent reviews have not supported routine OAC after MI in patients with reduced LVEF who remain in sinus rhythm without documented LV thrombus.^9^^,^^19^^,^^20^ In our study, OAC was associated with lower stroke risk only among patients with AF. Atrial fibrillation was the strongest and most consistent predictor of ischaemic stroke. Patients with AF had more than a 2-fold higher risk after adjustment for age, sex, LVEF and other comorbidities, consistent with previous evidence that AF-related strokes may occur later after MI and may be more severe.^21–24^ The significant AF–OAC interaction in our study indicates that the association between OAC and stroke differed according to AF status, aligning with established evidence supporting OAC for stroke prevention in AF.^25–27^

In contrast, no association between OAC and lower stroke risk was observed among patients without AF. This may partly reflect confounding by indication, as OAC recipients may have been older, had reduced LVEF or had other conditions associated with higher baseline risk. Although current guidelines do not recommend OAC in patients with sinus rhythm without AF or documented LV thrombus,^28^^,^^29^ 31.7% of patients receiving OAC in our study had no documented AF. This may reflect residual clinical uncertainty, earlier evidence supporting warfarin in patients with heart failure and sinus rhythm^30^^,^^31^ or other indications that are not fully captured in our data. Oral anticoagulant persistence at one year was substantially lower among non-AF than AF patients (35.5% vs 59.1%), suggesting less sustained treatment exposure in the non-AF group, although persistence cannot be equated with adherence.^32^ In the WARCEF trial, warfarin reduced ischaemic stroke compared with aspirin among patients with LVEF ≤ 35% in sinus rhythm, but increased major bleeding and provided no overall net benefit for the primary composite outcome,^31^ supporting current recommendation against routine OAC use in this setting.^20^^,^^30^^,^^31^^,^^33–35^ Thus, our findings do not provide evidence to support OAC use solely based on previous MI or reduced LVEF without AF or other established indication.

For intracerebral haemorrhage, the lower sHR associated with the AF × OAC interaction at 5 years and with antiplatelet therapy was unexpected, as antithrombotic treatment is generally associated with concern regarding intracranial bleeding.^36^^,^^37^ These findings should be interpreted cautiously given the relatively small number of intracerebral haemorrhage events, the observational design and the possibility of residual confounding and treatment selection. Therefore, these associations should not be interpreted as evidence of a protective effect of antithrombotic treatment against intracerebral haemorrhage.

Reduced LVEF was independently associated with ischaemic stroke, with progressively higher risk with worsening LV systolic function, consistent with previous evidence linking heart failure and LV dysfunction to increased stroke risk in sinus rhythm,^15^^,^^38^^,^^39^ likely through mechanisms such as blood stasis, endothelial injury and inflammation.^40^ These findings suggest that reduced LVEF may help identify patients with residual stroke risk after MI, particularly among those who remain in sinus rhythm. Further research is needed to determine whether improved risk stratification, detection of LV thrombus and optimisation of heart failure management can better identify patients who may benefit from targeted stroke-prevention strategies.

### Strengths and limitations

This study has several notable strengths. It leverages high-quality nationwide data from the SWEDEHEART registry, which provides comprehensive, clinical and follow-up information on a large, unselected cohort of MI survivors in Sweden. The linkage of multiple national registries enabled exploration of stroke outcomes and detailed adjustment for demographic, clinical and psychosocial variables. The large sample size and long follow-up period allowed for stratified competing risk analyses, enhancing the generalisability and clinical relevance of our findings. In addition, the use of competing risk analyses provides a more accurate estimation of stroke risk in the presence of the substantial competing risk of death as well as different stroke subtypes in this population.

Nonetheless, several limitations should be acknowledged. SWEDEHEART primarily registers patients below 80 years of age at the index MI. The incidence estimates may not accurately reflect absolute stroke risk in older MI survivors, in whom stroke risk is generally higher. Furthermore, LVEF was assessed during the acute MI hospitalisation and may have changed by the 6- to 10-week follow-up; therefore, it may not reflect cardiac function at the start of follow-up. Also, associations were adjusted for covariates at baseline, overlooking time-varying factors.

Another limitation is that detailed information on ischaemic stroke subtypes was incomplete, therefore, analyses differentiating specific stroke subtypes could not be performed. In addition, due to the observational design, we cannot draw causal conclusions, and potential residual confounding may exist due to the limited availability of variables. Medication (eg, OAC, statins, antiplatelets) and participation in components of comprehensive CR were not randomised and were recorded only at baseline or the 6- to 10-week follow-up, limiting causal inference and raising the possibility of confounding by indication. Indications for OAC therapy are not registered in SWEDEHEART. Anticoagulant therapy in patients without AF may thus reflect clinical judgement in response to other unmeasured risks and/or co-morbid conditions with OAC indications, which could bias associations.

## Supplementary Material

Supplemental_Material_aakag138

## Data Availability

Data supporting our findings can be obtained by contacting K.S.S. According to Swedish regulations (epn.se/en/start/regulations), permission to use data can only be based on application and approval from the Swedish Ethical Review Author.

## Trial registration

Not applicable because this was an observational registry-based study and not a clinical trial.
