# Supplementary material for: Ischaemic stroke and intracerebral haemorrhage in the post-acute phase after myocardial infarction: a nationwide cohort study in Sweden
Source: Eur Stroke J. 2026 Nov 12;11(11):aakag138. doi: 10.1093/esj/aakag138 (PMC13755349; doi:10.1093/esj/aakag138)
Supplement: Supplementary file 1 [file supplemental_material_aakag138.zip › Supplemental Material Table_clean.docx]

**Supplement Table**

**eTable 1. International Statistical Classification of Diseases and Related Health Problems 10th Revision codes used in this study.**

| **Comorbidities** | **ICD-10 code(s)** |
| --- | --- |
| Stroke | I61, I63 |
| Ischemic stroke | I63 |
| Intracerebral hemorrhage | I61 |
| Diabetes mellitus | E10–E14 |
| Atrial fibrillation and flutter | I48 |
| Hypertension | I10–I15 |
| Hyperlipidemia | E78 |
| Alcohol overconsumption | F10 |
| Overweight | E65–E68 |
| Arteries diseases | I70–I79 |
| Heart failure | I50 |
| Renal dysfunction | N00–N19 |
| ***Abbreviation****: ICD-10, International Classification of Diseases, 10th Revision.* | |

**eTable 2. Distribution of patients with OAC after MI**

| **Variable** | **Value** | **Without OAC** | | **With**  **OAC** | | **p-value** |
| --- | --- | --- | --- | --- | --- | --- |
|  |  | n | % | n | % |  |
| **AF** | AF | 3,711 | 4.8% | 4,978 | 68.3% | <0.001 |
|  | No AF | 73,839 | 95.2% | 2,307 | 31.7% |  |
| **Age group (years)** | ≤50 | 6,519 | 8.4% | 210 | 2.9% | <0.001 |
|  | 51–60 | 18,239 | 23.5% | 793 | 10.9% |  |
|  | 61–70 | 31,572 | 40.7% | 2,643 | 36.3% |  |
|  | 71–80 | 20,763 | 26.8% | 3,554 | 48.8% |  |
|  | >80 | 457 | 0.6% | 85 | 1.2% |  |
| **CHA₂DS₂-VASc score** | 1 | 12,676 | 16.3% | 318 | 4.4% | <0.001 |
|  | 2 | 22,697 | 29.3% | 1,222 | 16.8% |  |
|  | 3 | 21,959 | 28.3% | 2,118 | 29.1% |  |
|  | 4 | 13,775 | 17.8% | 2,010 | 27.6% |  |
|  | 5 | 5,195 | 6.7% | 1,177 | 16.2% |  |
|  | 6 | 1,155 | 1.5% | 391 | 5.4% |  |
|  | 7 | 93 | 0.1% | 49 | 0.7% |  |
| **Stroke Event** | No Stroke | 73,807 | 95.2% | 6,846 | 94.0% | <0.001 |
|  | Ischemic | 3,289 | 4.2% | 390 | 5.4% |  |
|  | Hemorrhagic | 454 | 0.6% | 49 | 0.7% |  |
| **LV Function** | Normal (≥50%) | 45,899 | 67.7% | 3,091 | 47.8% | <0.001 |
|  | Slightly reduced (40-49%) | 14,005 | 20.7% | 1,627 | 25.2% |  |
|  | Moderately reduced (30-39%) | 6,291 | 9.3% | 1,162 | 18.0% |  |
|  | Severely reduced (<30%) | 1,573 | 2.3% | 586 | 9.1% |  |
|  |  |  |  |  |  |  |

*A total of 525 patients with missing information on OAC use were excluded from the analysis.*
***Abbreviations****: AF, atrial fibrillation; CHA₂DS₂-VASc, congestive heart failure, hypertension, age ≥75 years, diabetes mellitus, stroke, vascular disease, age 65–74 years, and sex category; LV, left ventricular; OAC, oral anticoagulant.*

**eTable 3. Comparison between prescribed and dispensed oral anticoagulant therapy after myocardial infarction.**

|  | **SWEDEHEART-CR** | **Swedish prescribed drug register** | | | | |
| --- | --- | --- | --- | --- | --- | --- |
| **AF** | **OAC use prescribed at 1^st^ MI Follow Up (n)** | **0m (n, %)** | **3m (n, %)** | **6m (n, %)** | **1y (n, %)** | **5y (n, %)** |
| Yes | 4,978 | 3,374 (67.8%) | 3,156 (63.4%) | 2,980 (59.9%) | 2,944 (59.1%) | 1,395 (28.0%) |
| No | 2,307 | 1,480 (64.2%) | 1,075 (46.6%) | 896 (38.8%) | 819 (35.5%) | 432 (18.7%) |
| **Reduced LVEF** | | |  |  |  |  |
|  | 3375 | 2,612 (77.4%) | 2,336 (69.2%) | 2,180 (64.6%) | 2,129 (63.1%) | 1,048 (31.1%) |
| No | 3091 | 2,242 (72.5%) | 1,895 (61.3%) | 1,696 (54.9%) | 1,634 (52.9%) | 779 (25.2%) |
| *OAC use among patients with and without atrial fibrillation (AF) or reduced left ventricular ejection fraction (LVEF), recorded at the 6-10-week follow-up visit (SWEDEHEART-CR registry data) and tracked over time (0 m, 3 m, 6 m, 1 y, 5 y) using the Swedish Prescribed Drug Register (±30 days). Percentages indicate the proportion of patients with OAC use at the 6-10-week follow-up visit who also had a dispensed OAC prescription at each time point.*  ***Abbreviations****: AF, atrial fibrillation; LVEF, left ventricular ejection fraction; MI, myocardial infarction; OAC, oral anticoagulant; SWEDEHEART-CR, Swedish Web-system for Enhancement and Development of Evidence-based Care in Heart Disease Evaluated According to Recommended Therapies–Cardiac Rehabilitation.* | | | | | | |

**eTable 4. Stroke occurrence according to OAC type and AF status**

| **AF status** | **OAC type** | **Total, n** | **Stroke, n (%)** |
| --- | --- | --- | --- |
| AF | DOAC | 2,434 | 83 (3.4) |
|  | VKA | 2,538 | 229 (9.0) |
| No AF | DOAC | 606 | 22 (3.6) |
|  | VKA | 1,684 | 126 (7.5) |
| ***Abbreviations:*** *AF, atrial fibrillation; DOAC, direct oral anticoagulant; OAC, oral anticoagulant; VKA, vitamin K antagonist.* | | | |
