# Supplementary material for: Ischaemic stroke and intracerebral haemorrhage in the post-acute phase after myocardial infarction: a nationwide cohort study in Sweden
Source: Eur Stroke J. 2026 Nov 12;11(11):aakag138. doi: 10.1093/esj/aakag138 (PMC13755349; doi:10.1093/esj/aakag138)
Supplement: Supplementary file 1 [file supplemental_material_aakag138.zip › Supplemental Material Figure final.docx]

**Supplement Figure**

|  |  |
| --- | --- |
|  |  |
|  | |

**eFigure 1. Cumulative incidence of ischemic stroke stratified by key factors at the 6 to10 week follow-up visit after myocardial infarction.** Cumulative incidence was estimated using the Kaplan–Meier method. Thick lines represent point estimates, and shaded areas indicate 95% confidence intervals.

|  |  |
| --- | --- |
|  |  |
|  | |

**eFigure 2. Cumulative incidence of intracerebral hemorrhage stratified by key factors at the 6 to 10 week follow-up visit after myocardial infarction.** Cumulative incidence was estimated using the Kaplan–Meier method. Thick lines represent point estimates, and shaded areas indicate 95% confidence intervals.
